# Relationship between Temperament, Depression, Anxiety, and Hopelessness in Adolescents: A Structural Equation Model

**DOI:** 10.1155/2011/160175

**Published:** 2011-07-21

**Authors:** Paolo Iliceto, Maurizio Pompili, David Lester, Xenia Gonda, Cinzia Niolu, Nicoletta Girardi, Zoltán Rihmer, Gabriella Candilera, Paolo Girardi

**Affiliations:** ^1^Department of Neurosciences, Mental Health and Sensory Functions, Suicide Prevention Center, Department of Psychiatry, Sant'Andrea Hospital, Sapienza University of Rome, 1035 Via di Grottarossa, 00189 Rome, Italy; ^2^McLean Hospital, Harvard Medical School, Belmont, MA 02478, USA; ^3^McLean Hospital, Department of Psychiatry, Harvard Medical School, The Richard Stockton College of New Jersey, Galloway, NJ 08205-9441, USA; ^4^Department of Clinical and Theoretical Mental Health, Faculty of Medicine, Kútvölgyi Clinical Center, Kútvölgyi út 4., Semmelweis University, 1125 Budapest, Hungary; ^5^Department of Neuroscience, Unit of Psychiatry, University of Rome “Tor Vergata”, 00137 Rome, Italy; ^6^Department of Psychiatry and Psychological Medicine, Sapienza University of Rome, 00185 Rome, Italy; ^7^Private Practice, Rome, Italy

## Abstract

The purpose of this study was to test the validity of affective temperaments for predicting psychiatric morbidity and suicide risk, using a two-factor model to explain the relationships between temperament, anxiety, depression, and hopelessness. We investigated 210 high school students, 103 males and 107 females, 18-19 years old, who were administered self-report questionnaires to assess temperament (TEMPS-A), depression (BDI-II), anxiety (STAI) and hopelessness (BHS). The final structural model had a good fit with the data, with two factors significantly correlated, the first labeled unstable cyclothymic temperament including Dysthymic/Cyclothymic/Anxious temperament, Irritable temperament and Depression, and the second labeled Demoralization including Anxiety (State/Trait) and Hopelessness. Depression, anxiety and hopelessness are in a complex relationship partly mediated by temperament.

## 1. Background

The widely accepted etiological hypothesis proposes a cooccurrence between depression and anxiety, since these conditions share several symptoms and causal factors. However, hopelessness appears to play a unique role in this cooccurrence [1, 2]. 

It is of special importance to understand the mechanisms involved in the development of depression and anxiety in adolescents, not only because of the high rates of prevalence of these disorders all over the world [3] but also because this cooccurrence often increases the likelihood that adolescents will develop feelings of hopelessness [4] which is known to have links with suicidal ideation and suicidal behaviors [5–7]. 

Another important construct, demoralization, was described by Frank [8] as different from depression. Individuals who are demoralized are conscious of their own failure and are unable to meet the expectations of others. When their anxiety levels increase, they are likely to develop feelings of hopelessness and a desire to die [9]. The validity of the distinction between depression and demoralization is further supported by research showing that suicidal ideation is differentially associated with hopelessness and depression. Studies in adolescents have also shown that depression and hopelessness are independent predictors of suicidal ideation [10, 11]. 

Akiskal [12] has proposed that dysregulation of temperament is the fundamental pathology underlying mood disorders, and its presence in individuals reflects an increased predisposition for developing affective disorders. Moreover, specific affective temperament types (depressive, cyclothymic, hyperthymic, irritable, and anxious) have a strong relationship with suicidal behavior [13], and a study regarding affective temperaments in suicide attempters indicated that compared to controls suicide attempters scored significantly higher on four of the five affective temperaments containing a more or less depressive component [14]. 

In spite of the fact that all the above factors, depression, anxiety, hopelessness, and affective temperaments, are related to suicidal behavior, no study so far investigated the interrelationship between these phenomena in a single framework. Delineating the associations between these phenomena is crucial in understanding how they mediate and influence one another in the development of suicidal behavior, describing possibilities for intervention on multiple levels. The aim of our present study was to explore the association of the five affective temperaments in the Akiskal model (Depressive, Hyperthymic, Cyclothymic, Irritable, and Generalized Anxious) with anxiety, depression, and hopelessness in a sample of adolescents. Our study also extends the model of demoralization by introducing temperament dysregulation, and we propose a two-factor model that explains the relationships between temperament, anxiety, depression, and hopelessness.

## 2. Method

### 2.1. Participants

In the second half of 2009, 210 adolescents from the south of Italy voluntarily participated in this study. The participants were high school students, aged 18 to 19 years, 103 males (mean age: 18.43, SD:  .49) and 107 females (mean age: 18.49, SD:  .50). All students were from the same grade school and were late adolescents. After obtaining the permission from administrators of the schools, we administered four psychometric instruments. All subjects were culturally homogeneous, although they came from families with various socioeconomic backgrounds (including farmers, merchants, professionals, and industry workers), mainly middle class to upper-middle class. Their sociodemographic characteristics are shown in Table 1. Subjects participated voluntarily in the study, and each subject provided written informed consent. The study protocol received ethics approval from the local research ethics review board.

### 2.2. Instruments

The first version of *Temperament Evaluation of Memphis, Pisa, Paris and San Diego* contained 84 items [15]. Later, clinical and theoretical considerations led to the addition of 26 new items describing the anxious temperament, resulting in the 110-item-long version of the TEMPS-A [16]. The scale is different from most other temperament scales in that it taps subaffective trait expressions as they were conceptualized in Greek psychological medicine and, in more modern times, German psychiatry. The TEMPS-A has been validated in Italian populations [17]. In that investigation, a principal-component analysis with a varimax rotation resulted in a 3-factor solution. The first (25.5% of the variance) contained the dysthymic, cyclothymic, and anxious (Dys/Cyc/Anx) temperaments combined, the second the irritable temperament (7.5% of variance), and the third the hyperthymic temperament (4.4% of variance). Kuder-Richardson reliability coefficients ranged from 0.90 for the Dys/Cyc/Anx factor to 0.76 for the hyperthymic factor.

The *Beck Hopelessness Scale *[18, 19] is a 20-item scale for measuring negative attitudes about the future. Beck originally developed this scale in order to predict who would commit suicide and who would not. This powerful predictor of eventual suicide addressed three major aspects of hopelessness: feelings about the future, loss of motivation, and expectations. Responding to the 20 true or false items on the BHS, individuals can either endorse a pessimistic statement or deny an optimistic statement. Research consistently supports a positive relationship between BHS scores and measures of depression, suicidal intent, and current suicidal ideation. In addition, Beck et al. [20] carried out a prospective study of 1,958 outpatients and found that hopelessness, as measured by the BHS, was significantly related to eventual suicide. A cutoff score of 9 or above identified 16 (94%) of the 17 patients who eventually committed suicide. According to that study, the high-risk group identified by this cutoff score was 11 times more likely to commit suicide than the rest of the outpatients. The BHS may, therefore, be used as a proxy indicator of suicide potential. Studies have been carried out indicating its validity for an Italian population [21, 22].

The *Spielberger State Trait Anxiety Inventory *[23] is a 40-item self-report measure of enduring (trait) and transient (state) anxiety symptoms. Respondents rate how statements reflect how they generally feel on a 4-point scale. STAI state and trait scores range from 20 to 80. State anxiety is defined as unpleasant emotional arousal, characterized by feelings of tension, apprehension, and heightened autonomic nervous system activity, while trait anxiety measures a stable tendency to respond with state anxiety [24].

The *Beck Depression Inventory Second Edition *[25] is a 21-item self-report measure of symptoms of depression. Respondents choose statements that reflect how they have felt over the past 2 weeks. The BDI contains 13 items assessing cognitive-mood depressive symptoms (e.g., sadness, guilt) and 8 items that assess physical symptoms of depression (e.g., fatigue, weight loss, and physical health worries).

### 2.3. Statistical Analyses

Two-tailed *t*-tests and Pearson correlations were used for continuous variables and chi-square test with Yates's correction where appropriate to identify differences in socio-demographic characteristics. All analyses were carried out using SPSS 17.0. The hypothesized structural relations in the model were assessed by the means of structural equation modeling with the use of AMOS 16.0. 

Structural Equation Modeling (SEM) relies on several statistical tests to determine the adequacy of model fit to the empirical data. Specifically, confirmatory factor analysis (CFA) allows testing a hypothesis concerning a relationship between the observed variables and their underlying latent constructs. In this way, it is possible to use the prior empirical research to postulate a relationship pattern *a priori* and then test the hypothesis statistically. CFA seeks to determine if the number of factors and the loadings of the measured (indicator) variables on the factors conform to what is expected on the basis of preestablished theory and research results. Indicator variables are selected on the basis of prior theory, and factor analysis is used to see if they load as predicted on the expected number of factors. The researcher's *a priori* assumption is that each factor is associated with a specified subset of indicator variables. A CFA requires the formal specification of the measurement instrument in terms of a factor model, the statistical fitting of the factor model to the observed data (variances and covariances or correlations), the assessment of fit, and the interpretation of the results if the model is consistent with the data [26].

The chi-square test indicates the amount of difference between the expected and observed covariance matrices. A chi-square value close to zero indicates little difference between the expected and observed covariance matrices. In addition, the probability level must be greater than 0.05 when chi-square is close to zero. The Comparative Fit Index (CFI) is equal to the discrepancy function adjusted for sample size. The CFI ranges from 0 to 1 with a larger value indicating better model fit. Acceptable model fit is indicated by a CFI value of 0.90 or greater. Root Mean Square Error of Approximation (RMSEA) is related to the residual in the model. RMSEA values range from 0 to 1 with a smaller RMSEA value indicating better model fit. Acceptable model fit is indicated by an RMSEA value of 0.06 or less [27, 28].

## 3. Results

The descriptive statistics and zero-order correlations between the variables are presented in Table 2. All the correlations are consistent with the results in the empirical literature.

Two confirmatory factor models were specified using the sample covariance matrix and the estimated parameters using maximum likelihood method. Model 1 was a 1-factor model with all the six variables loading on a general factor. Model 2 represented a two-factor model with three variables loading on each of their respective factors, and these were as hypothesized. The first model was statistically overidentified and produced fit indices as follows: *χ* ^2^(9)  = 47.33 (*P* < .000); *χ* ^2^/DF = 5.25; CFI = .95; RMSEA = .14, with all moderate loadings. The hypothesis that this model is a good fit to the data is easily rejected.

**Figure 1:**
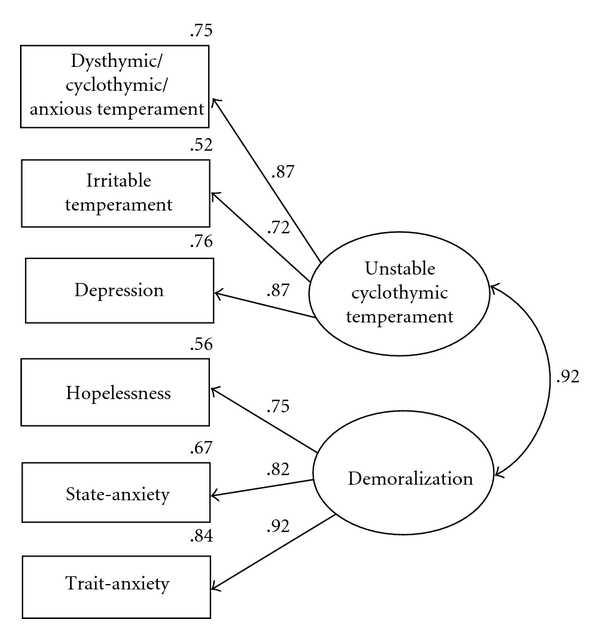
Structural Equation Model with two correlated factors. The curved arrow represents the relationship between the latent factors, while straight arrows from latent factors to observed variables represent factor loadings.

It seemed likely, therefore, that a two-factor model is more appropriate to describe the relationships between observed variables and latent factors, and this model appears to fit the data substantially better than the single factor model. The second model produced fit indices as follows: *χ* ^2^(8) = 29.97 (*P* < .000); *χ* ^2^/DF = 3.75; CFI = .97; RMSEA = .05. Although this analysis yielded a statistically significant chi-square statistic, the inspection of comparative fit indexes and a lower RMSEA revealed that there was a good fit between the hypothesized model and the data. The inspection of model parameters indicated that all the variables significantly loaded on the appropriate factor and that the factors are significantly correlated (*r* = .92). The Dysthymic/Cyclothymic/Anxious temperament, the Irritable temperament, and depression had strong loadings on a factor which can be labelled Unstable cyclothymic temperament (.87,  .72, 87, resp.), whereas Hopelessness, State-Anxiety and Trait-Anxiety had strong loadings on a factor which may be labelled Demoralization (.75,  .82,  92, resp.) (see Figure 1). The squared multiple correlation coefficients (*R* ^2^) describe the amount of variance the common factor accounts for in the observed variables and, in order of increasing magnitude, Unstable cyclothymic temperament explains about 52% of the variance of Irritable temperament, 75% of Dysthymic/Cyclothymic/Anxious temperament, and 76% of Depression, whereas Demoralization explains about 56% of the variance of Hopelessness, 67% of State-Anxiety, and 84% of Trait-Anxiety. Thus, we conclude that the two-factor model with correlated factors provided an acceptable fit to the empirical data. 

## 4. Discussion

This study sought to test two models for explaining the relationship between anxiety, depression, hopelessness, and affective temperaments The novelty of the present study, compared to that of Cunningham and colleagues [29], was to introduce the Dysthymic/Cyclothymic/Anxious temperament and Irritable temperament as observed variables in addition to those already identified in previous research—anxiety (state/trait), depression, and hopelessness. The results indicated a distribution of the six observed variables into two latent factors, the first labeled unstable cyclothymic temperament, representing the link between affective temperaments and depression, and the second factor labeled Demoralization, involving anxiety (state/trait) and hopelessness, but not depression. 

The unstable cyclothymic temperament factor included the Irritable temperament (52%) and the Dysthymic/Cyclothymic/Anxious temperament (75%), as well as the component of depression (76%), involving individuals' inability to experience positive emotions, feeling tired and dissatisfied, having a sensory response that involves slowing down, and holding general critical thoughts about oneself. Depression was associated with a feeling of vulnerability and structurally characterized by specific temperament traits with negative consequences both on personal mood and on interpersonal relationships. Thus the Unstable cyclothymic temperament factor involved a psychological condition in which individuals perceive a state of disequilibrium, characterized by generally pessimistic and self-critical cognitions. 

The second factor, Demoralization, involved the constructs of Trait Anxiety (84%), State Anxiety (67%) and Hopelessness (56%) and, as with the previous factor, there were sensory and cognitive components. In this case, the “feeling” has no direct consequence with the representation of themselves as people with a basic structure of personality that is able or unable to act or think, but shifts attention from the here and now (I am) to a representation of a future that is negative and unchangeable. Hopelessness and anxiety are associated with a temporal perspective of negative future expectations and suicidal ideation. 

Incidentally, previous research has shown that the Dysthymic/Cyclothymic/Anxious temperament predicts hopelessness but, unexpectedly, depression is not a good predictor of the risk of suicide [30].

The strength of the present study is that it has revealed the relevance of what has been called Unstable cyclothymic temperament, a latent factor that involves affective temperaments and depression, in addition to Demoralization that consists of anxiety and hopelessness. It extends the theoretical framework and provides a new approach for clinical intervention in the treatment of symptoms of depression and anxiety in adolescents and for preventing suicidal behaviors. 

The novelty of our study is the introduction of affective temperaments in the model of phenomena closely related to and possibly predicting suicidal behavior. Affective temperaments are a crucial part of this model because due to their spectrum nature they underlie normal healthy personality processes, present a latent form of affective pathology and predispose to affective illness, and have a pathoplastic role in case of major mood disorders as well. The overall finding of our study establishes a general framework of those psychologically relevant factors known to play a crucial role in the background of suicidality. Better understanding of how depression, anxiety, hopelessness, and affective temperaments relate to each may provide us with a model for understanding the evolution of suicidal behavior as well as helping us determine multiple points of intervention at multiple levels.

However, there were some limitations to this study, such as a cross-sectional study design, while longitudinal studies are more informative; moreover the limited sample size (making exploration of sex differences in these associations impossible) and the use of a nonclinical population hinder the generalization of the results. 

Furthermore, psychometric instruments used in this paper were used in a non-clinical population rather than a clinical population whereas they were involved in many comprising clinical populations. Therefore, our study lacks information related to lifetime depression or hypomania; in addition it lacks self-rated scale for hypomania, as well as data concerning the psychological role of hyperthymic temperament, and scales exploring the attitudes, beliefs, and cognitions. 

Despite these caveats, this paper offers an original perspective that may be of help in understanding complex relationship between constructs involved in our study. Further research is needed on how temperament and hopelessness may be related to the cooccurrence of depressive and anxiety symptoms. There may be other pathways involved in these links, such as emotional distress, mood disorders, and insecure attachment. Research should also investigate the role of cognitions and expectations of others in interpersonal contexts on these associations.

## Figures and Tables

**Table 1:** Socio-demographic characteristics of subjects.

Characteristics	Males (*N* = 103)	Females (*N* = 107)	Statistics	*P*
Age (years)	18.43 ± .49^a^	18.49 ± .50^a^	*t*_(208)_ = .85	.39
Sociocultural level of families			*χ*_(2)_^2^ = 5.90	.05
Low (N)	9	14		
Middle (N)	69	54		
High (N)	25	39		

^a^ Values shown as mean ± SD.

**Table 2:** Descriptive statistics and zero-order correlations.

	Means	SD	2	3	4	5	6
(1) TEMPS-A—Dys/Cyc/Anx	18.89	7.97	.605	.579	.748	.716	.736
(2) TEMPS-A—Irritable	6.39	3.28		.470	.671	.589	.571
(3) BHS	6.23	4.99			.634	.544	.717
(4) BDI-II	15.80	10.96				.648	.719
(5) STAI-S	48.55	13.30					.752
(6) STAI-T	46.89	11.63					

*Note*. All correlations significant at *P* < .001; TEMPS-A: Temperament Evaluation of Memphis, Pisa, Paris and San Diego; BHS: Beck Hopelessness Scale; BDI-II: Beck Depression Inventory Second Edition; STAI: Spielberger State Trait Anxiety Inventory.
